# An Unexpected Location of the Arginine Catabolic Mobile Element (ACME) in a USA300-Related MRSA Strain

**DOI:** 10.1371/journal.pone.0016193

**Published:** 2011-01-25

**Authors:** Mette Damkjær Bartels, Lars Hestbjerg Hansen, Kit Boye, Søren J. Sørensen, Henrik Westh

**Affiliations:** 1 Department of Clinical Microbiology, Hvidovre Hospital, Hvidovre, Denmark; 2 Department of Biology, University of Copenhagen, Copenhagen, Denmark; 3 Faculty of Health Sciences, University of Copenhagen, Copenhagen, Denmark; National Institutes of Health, United States of America

## Abstract

In methicillin resistant *Staphylococcus aureus* (MRSA), the arginine catabolic mobile element (ACME) was initially described in USA300 (t008-ST8) where it is located downstream of the staphylococcal cassette chromosome *mec* (SCC*mec*). A common health-care associated MRSA in Copenhagen, Denmark (t024-ST8) is clonally related to USA300 and is frequently PCR positive for the ACME specific *arc*A-gene. This study is the first to describe an ACME element upstream of the SCC*mec* in MRSA. By traditional SCC*mec* typing schemes, the SCC*mec* of t024-ST8 strain M1 carries SCC*mec* IVa, but full sequencing of the cassette revealed that the entire J3 region had no homology to published SCC*mec* IVa. Within the J3 region of M1 was a 1705 bp sequence only similar to a sequence in *S. haemolyticus* strain JCSC1435 and 2941 bps with no homology found in GenBank. In addition to the usual direct repeats (DR) at each extremity of SCC*mec*, M1 had two new DR between the *orf*X gene and the J3 region of the SCC*mec*. The region between the *orf*X DR (DR1) and DR2 contained the *ccr*AB4 genes. An ACME II-like element was located between DR2 and DR3. The entire 26,468 bp sequence between DR1 and DR3 was highly similar to parts of the ACME composite island of *S. epidermidis* strain ATCC12228. Sequencing of an ACME negative t024-ST8 strain (M299) showed that DR1 and the sequence between DR1 and DR3 was missing. The finding of a mobile ACME II-like element inserted downstream of *orf*X and upstream of SCC*mec* indicates a novel recombination between staphylococcal species.

## Introduction

The arginine catabolic mobile element (ACME) has been described in both coagulase negative staphylococci and MRSA. The prevalence is high in *S. epidermidis* and *S. haemolyticus* [1], [2] where it is found in several different genetic backgrounds, whereas it has only been described in a few MRSA lineages [3]. ACME in MRSA has mostly been described in the USA300 clone, where it is located downstream of the staphylococcal cassette chromosome *mec* (SCC*mec*) [4]. The prototype USA300 (USA300-0114) belongs to sequence type (ST) 8, is *spa* type t008 and harbours SCC*mec* IVa [5]. The most common healthcare associated MRSA in Copenhagen, Denmark is t024-ST8-IVa [6] and by pulsed-field gel electrophoresis (PFGE) the t024 clone cannot be discriminated from USA300 [7]. Furthermore, most t024 isolates are PCR positive for the ACME-specific *arc*A gene allele. We have previously shown that the BD GeneOhm MRSA assay failed to detect most of our t024 isolates [8]. While all eleven t008-IVa isolates in the study were positive by the BD GeneOhm MRSA assay, this was the case for only five of thirty-three t024-IVa isolates and those five isolates were PCR positive in a late cycle indicating a false positive result. These results indicated that the J3 region of SCC*mec* IVa in t008 and t024 were not identical. In an attempt to study this phenomenon we discovered that PCR in the *orfX* region yielded no products by long range PCR and that the t024 isolates lacked the 2 kb downstream constant segment (*dcs*) [9] found in the J3 region of most SCC*mec* IV subtypes [10]. This suggested substantial changes in the J3 region and prompted us to sequence the entire genome of one ACME-positive and one ACME-negative t024 isolate. In this paper the SCC*mec* and the *orf*X near region of these two isolates are characterized.

## Materials and Methods

Strains: From a collection of 311 t024 isolates (88 % PCR positive for the ACME specific *arc*A gene allele) we selected two MRSA strains (M1 and M299) that were t024-ST8-IVa and Panton-Valentine leukocidin (PVL) negative. M1 was PCR positive for the ACME-specific *arc*A gene allele [4] and M299 was PCR negative. The isolates were healthcare associated (HCA)-MRSA isolated in 2003 and 2005, respectively and had been stored at minus 80 degrees.

Typing and sequencing: *spa* typing [6], SCC*mec* typing [11], [12], MLST [6], PCR for PVL [6] and ACME [4] were performed as previously described. DNA was extracted from colonies on 5 % blood agar plates (Statens Serum Institut, Copenhagen, Denmark) using the Ultraclean microbial DNA isolation kit (MO BIO Laboratories Inc., Carlsbad, CA, USA). DNA concentration was measured on a NanoDrop 1000 spectrophotometer (Thermo Scientific, Wilmington, DE, USA). Whole genome sequencing was performed on a GS FLX (454 Life Sciences, a Roche company, CT, USA). For the M1 strain approximately two µg DNA was used to build a single stranded (ss) library according to GS FLX Library preparation manual (Roche). The ssDNA library was quantified by qPCR using primers targeting the A & B adaptors. Also a ss-DNA standard of 180 bases in length and Brilliant® SYBR® Green QPCR Master mix (Stratagene, Cedar Creek, TX, USA) were used. Test emulsion PCR's were performed to obtain the best copies/ bead ratio. DNA containing beads were sequenced using the LR70 sequencing kit on a full standard Pico Titre Plate (PTP). The M299 strain was sequenced using the GS FLX Titanium chemistry on one-quarter Titanium PTP. The ssDNA library of M299 was prepared from five µg DNA according to the GS FLX Titanium Library preparation protocol (Gel-cut method) and quantified and titrated similarly to the standard library by qPCR and test emulsions. Reads were aligned and assembled using the Newbler assembler software provided with the GS FLX instrument.

Because the SCC*mec* and *orf*X near sequences were on more than one contig, sequence-specific primers were designed for gap closure and PCR products were sequenced on an ABI 3130 XL (Applied Biosystems Inc, Foster City, CA, USA). Single-nucleotide polymorphisms (SNPs) between M1 and M299 were double-checked by standard PCR and sequencing. Sequences were compared to published sequences using BLAST (GenBank), and ORF Finder (GenBank) was used to identify possible open reading frames (ORFs) in cases where the sequence had no significant similarity to published sequences in GenBank.

### Nucleotide sequence accession numbers

The M1 and M299 SCC*mec* and *orf*X near sequences have been deposited in the GenBank/EMBL/DDBJ databases under accession no. HM030720 and HM030721, respectively.

## Results

Whole genome sequencing of M1 and M299 had a 40-fold and 20-fold coverage, respectively. In M1, the sequence from *orf*X to the right extremity junction of SCC*mec* was localized on two contigs and after gap closure the entire sequence was 53,864 bps of which the last 27,380 bps constituted the SCC*mec*. In M299, the SCC*mec* was adjacent to *orf*X. It was localized on seven contigs and after gap closure the entire sequence was 27,380 bps. M1 and M299 contained a type 2 *ccr* gene complex and a class B *mec* gene complex characteristic of SCC*mec* type IV [10].

**Figure 1:**
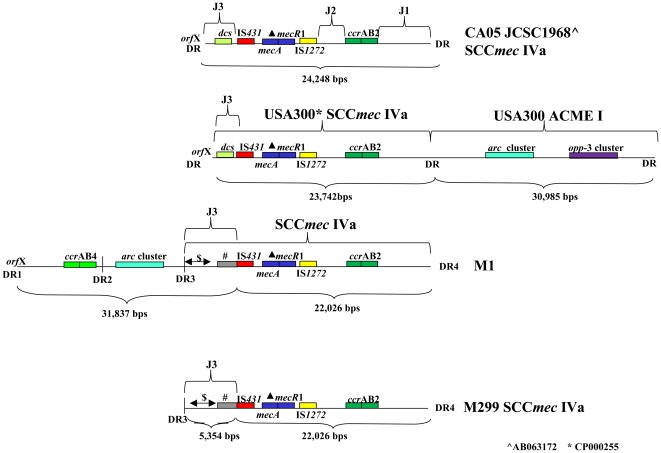
Comparison of SCC*mec* IVa and flanking regions of M1, M299, CA05 and USA300. Sequences of DR: DR1: GAAGCATATCATAAG. DR2: TGAAGCGTATCATAAGTGA. DR3: GGAAGCGTATCACAAATAA. DR4: AGAGGCGTATCATAAGTAA. The *arc* cluster includes the genes *arc*R, *arc*A,*arc*D, *arc*B and *arC*. $: 2941 bps with no significant similarity to sequences in Genbank. **#**: A sequence of 1705 bps only similar to *S. haemolyticus* strain JCSC 1435.

**Figure 2:**
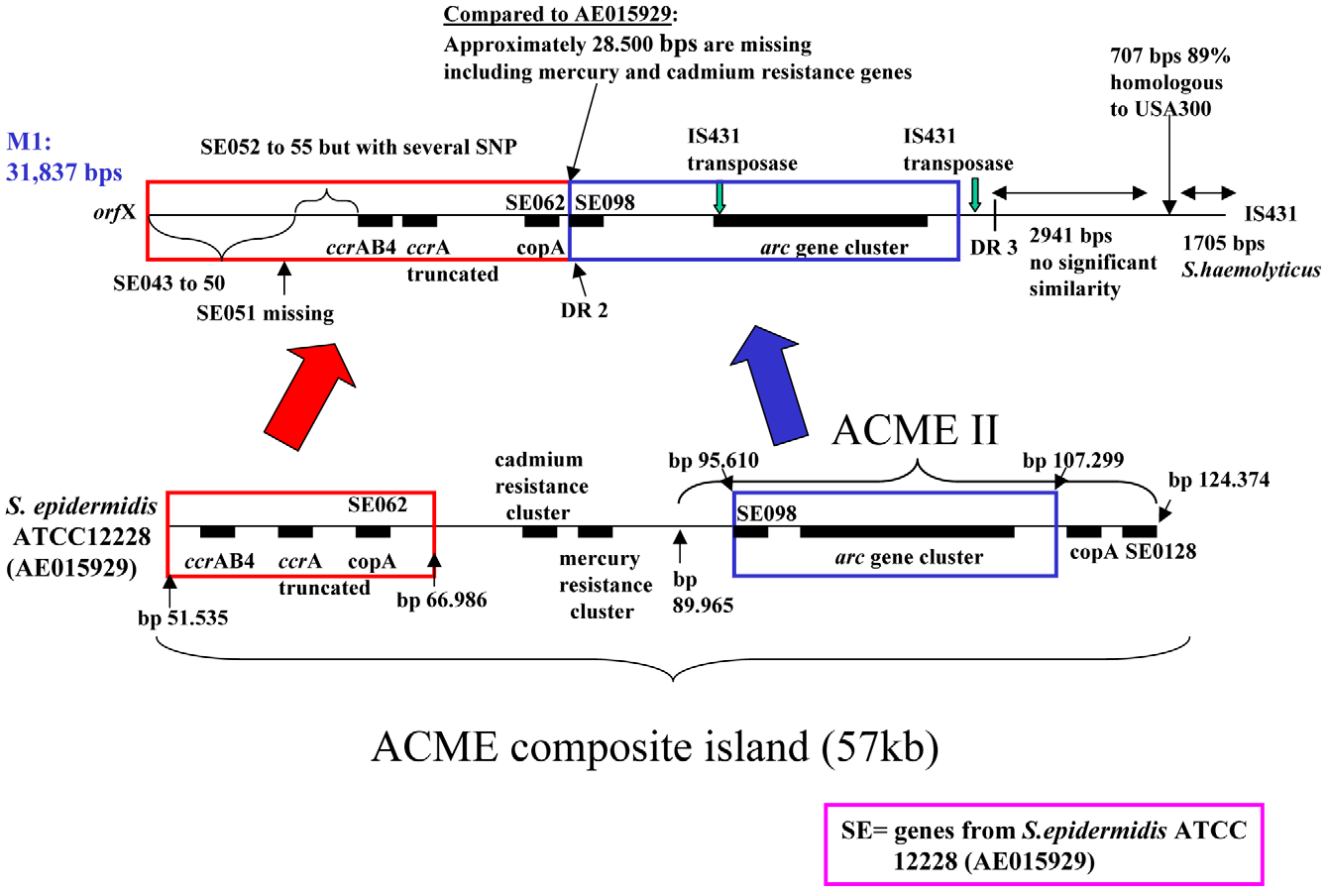
Comparison of the ACME composite island in *S. epidermidis* ATCC12228 (AE015929) and in M1.

In addition to the usual direct repeats (DR) at each extremity of SCC*mec* (DR1 and DR4 in Table S1 and Figure 1), two DR were identified in M1 (DR2 and DR3 in Table S1 and Figure 1). The entire sequence from DR1 to DR3 was highly similar to the ACME composite island of strain *S. epidermidis* ATCC12228 (AE015929) [4]. The sequence included the arginine deiminase pathway (*arc*) cluster and the *ccr*AB4 genes. However, compared to the ACME composite island of ATCC12228 it lacked about 28.5 kbs of centrally placed genes including the genes coding for mercury and cadmium resistance. In addition, the ACME II-like element of M1 was shorter than the ACME II described in *S. epidermidis* (Figure 2) M299 had no additional sequence between the *orf*X and SCC*mec*, but the SCC*mec* was identical to the M1 cassette and interestingly, it started with DR3 and not with the typical DR1 sequence (Figure 1).

The 5,354 bps from DR3 to the *mec* gene complex in M1 and M299 were considered to constitute the J3 region of their SCC*mec* (Table S1). The J3 region of M1 and M299 had no homology with published type IVa sequences (Figure 1). The first 2,941 of the 5,354 bps showed no significant similarity to GenBank sequences, but contained six possible ORFs. Four of these ORFs were by protein blast identical to *S. epidermidis* hypothetical proteins (Table S1) while two ORFs had no significant similarity to proteins in GenBank. The next 636 bps only showed similarity to USA300 FPR3757 and USA300 TCH1516 (89 % similarity). In USA300 this sequence included part of two genes located in the beginning of ACME I [4]. The following 71 bps were identical to a sequence in J1 of many MRSA and included part of a gene coding for a hypothetical protein. Finally, the remaining 1,705 bps were only similar to *S. haemolyticus* JCSC1435 (AP006716) (Table S1).

The sequence ranging from the beginning of the *mec* gene complex to the right extremity junction in M1 and M299 was identical in the two isolates and had only one SNP compared to the sequence of MRSA CA05 (JCSC1968) [13]. The SNP was in J1 in a non-coding region.

When comparing the SCC*mec* of M1 and M299, *i.e.* the sequence from DR3 to DR4 (Figure 1), the GS FLX sequencing found eleven possible SNPs. Six were in J1, two in the *mec* gene complex and three in J3. All but one SNP were in regions with homopolymers and were mainly missing nucleotides in M299. None of the eleven SNPs were found when standard PCR and sequencing were applied.

## Discussion

The present study revealed recombination events in the J3 region of two MRSA SCC*mec* IVa and the presence of a sequence highly similar to the ACME composite island of *S. epidermidis* ATCC12228 in one isolate. The 53,863 bp sequence ranging from *orf*X to the right extremity junction of SCC*mec* in M1 contained two *ccr* gene complexes (*ccr*AB2 and *ccr*AB4), one *mec* gene complex and an ACME II-like element. Two new DR were identified in M1 between DR1 and the J3 region of SCC*mec* IVa. Assuming that these DR are recognized by *ccr* genes, the sequence between *orf*X and the right extremity junction of SCC*mec* in M1 can be considered a composite element consisting of three parts. In agreement with the recently published guidelines for naming the SCC elements [10], we name the 53,863 bp composite element as follows: SCC*_M1_*; ψSCC*_arc;_* SCC*mec*IVa (2B); J1, subtype 1-specific ORFs; J3, subtype 3-specific ORFs for M1. The SCC*mec* of MRSA isolate M299 was 27,380 bp long and contained only one *ccr* gene complex (*ccr*AB2). The SCC*mec* of M299 was identical to the SCC*mec* IVa of M1.

Most of the sequence between DR1 and DR3 in M1 was highly homologous to the *S. epidermidis* ACME composite island in strain ATCC12228 (AE015929) [4] but interestingly the genes coding for mercury and cadmium resistance were not present in M1 (Figure 2). This could indicate that the genetic material acquired by M1 came from a *S. epidermidis* background with another variant of the ACME composite island, alternatively these resistance genes could have been lost in M1 because of a high fitness cost. The *arc* gene cluster in M1 was surrounded by sequences highly similar to the ones in *S. epidermidis* ATCC12228 so we presume that it originated from a *S. epidermidis* although ACME has also been found to be prevalent in *S. haemolyticus* [2]. Surprisingly, in M1, ACME was localized upstream of SCC*mec* and not downstream of SCC*mec* as in MRSA USA300 [4]. Therefore it would be interesting to identify the location of ACME in other non-USA300 MRSA isolates. Large SCC elements are usually believed to create low fitness, but the ACME-positive t024-ST8-IV clone has spread in nursing homes in Copenhagen since 2003 and only due to increased infection control and focus on this particular clone, the number of cases has decreased and the clone has been under control for the last couple of years. The ACME-negative M299 clone has been less successful. The first ACME-negative isolates were identified in 2005, two years after the ACME-positive clone appeared, and did not replace the ACME-positive clone (data not shown). Diep *et al* [4] have described ACME in USA300 and have shown in a rabbit model that it enhances fitness and pathogenicity of the clone [3], but this finding could not be confirmed in rat models [14]. Interestingly, a study by Miragaia *et al* [1] on ACME in *S. epidermidis* suggests that ACME leads to an enhanced colonization and transmission rather than increased pathogenicity. This is consistent with the fact that the ACME-positive t024-ST8-IVa clone spread easily in nursing homes and decolonization treatments often failed, and it might explain why the clone had success in spite of a large composite element. Since ACME-positive t024-MRSA appeared before ACME-negative t024-MRSA in Copenhagen, we presume that the *ccr* genes in M1 are responsible for the excision of 26,483 bps resulting in the M299 variant.

The 5,354 bps in J3 of M1 and M299 were an interesting mixture of 2,941 bps with no significant similarity to published sequences, 707 bps that were mostly similar to USA300 and 1,705 bps that were highly homologous to *S. haemolyticus*. The sequence without significant similarity had a GC content of 25 % and according to Takeuchi *et al*, low GC content is found in non-coding regions of Staphylococcal species [15]. Nevertheless, four possible ORFs in this sequence potentially coded for proteins similar to proteins found in *S. epidermidis*, indicating that this region might originate from a *S. epidermidis* not yet sequenced. Recently, Miragaia *et al* [1] described a Danish *S. epidermidis* strain (DEN077) containing both *ccr*AB4, *ccr*AB2 and ACME, but the location of ACME was not determined. The presence, in M1, of a composite element including 31,837 bps with apparent diverse genetic background indicates a high rate of genetic exchange and recombination between staphylococcal species which has also been found by others [16]–[18]. Whether the recombination events occurred in *S. aureus* or in coagulase negative staphylococci remains to be determined.

The eleven SNPs between M1 and M299 found by GS FLX sequencing were not reproduced by standard PCR and sequencing. Ten of the SNPs were in regions with homopolymers.

GS FLX errors in homopolymers is a well-known problem [19]. It is not surprising that all the errors were in M299 since sequencing of this strain had a coverage of 20 compared to 40 for the M1 strain.

The substantial changes found in J3 of our two isolates are missed by our routine SCC*mec* IV subtyping [12]. As a result, isolates that are considered to have identical SCC*mec* might have substantial differences that are unrecognized. In addition, the real time PCR assay BD GeneOhm MRSA amplifies in the J3 region and failed to detect the two isolates [8]. This illustrates a continuous need to update PCR based MRSA detection in the *orf*X/J3 region, as would be necessary to detect our dominant t024-ST8-IVa clone. The microevolution of SCC*mec* cassettes seem to be rather fast, and new primers that target changing J3 regions can only be designed after variant clones are identified and analysed.

In conclusion, we have found ACME to be located upstream of SCC*mec* in a USA300-related MRSA strain. Furthermore, we have identified a SCC*mec* IVa with an entire novel J3 region including a sequence from *S. haemolyticus*. Our data suggest that considerable genetic exchange occurs between staphylococcal species and can lead to diversity in the SCC*mec* that is only recognized by complete nucleotide sequencing.

## Supporting Information

Table S1 # Possible ORFs using ORF finder, Genbank. ∧ The gene with the highest identity is listed. Only in cases where genes from several isolates have the same identity, all are listed. *The last 5 bps of the gene are missing in the M1 sequence. ^1^Only the last 118 of 330 bps. ^2^Only the last 612 of 867 bps. ^§^Possible direct repeats.
(DOC)
