# Supplementary material for: An Unexpected Location of the Arginine Catabolic Mobile Element (ACME) in a USA300-Related MRSA Strain
Source: PLoS One. 2011 Jan 25;6(1):e16193. doi: 10.1371/journal.pone.0016193 (PMC3026799; doi:10.1371/journal.pone.0016193)
Supplement: Table S1 — # Possible ORFs using ORF finder, Genbank. ∧ The gene with the highest identity is listed. Only in cases where genes from several isolates have the same identity, all are listed. *The last 5 bps of the gene are missing in the M1 sequence. 1Only the last 118 of 330 bps. 2Only the last 612 of 867 bps. §Possible direct repeats. (DOC) [file pone.0016193.s001.doc]

TABLE S1. Open Reading Frames (ORFs) between the *orf*X gene and the *mec* gene complex of M1 and M299

| ORFs  M1 | Nucleotide position | ORFs M299 | Nucleotide position | Size (bp) | Nucleotide blast % identity | Gene^ | Protein blast % identity | Gene product/protein | Species | GenBank  Accession number |
| --- | --- | --- | --- | --- | --- | --- | --- | --- | --- | --- |
| DR_SCC | 1-15 |  |  | 15 |  |  |  |  |  |  |
| 1 | 119-259 |  |  | 141 | 100 % | SE0043 |  | Type I restriction enzyme R protein | *S. epidermidis* | AE015929 |
| 2 | 255-1454 |  |  | 1200 | 100 % | SE0044 |  | Probable specificity determinant HsdS | *S. epidermidis* | AE015929 |
| 3 | 1464-1712 |  |  | 249 | 100 % | SE0045 |  | Truncated  transposase | *S. epidermidis* | AE015929 |
| 4 | 1783-2277 |  |  | 495 | 100 % | SE0046 |  | Spermidine acetyltransferase | *S. epidermidis* | AE015929 |
| 5 | 2559-2684 |  |  | 126 | 100 % | SE0047 |  | Hypothetical protein | *S. epidermidis* | AE015929 |
| 6 | 2952-3359 |  |  | 408 | 100 % | SE0048 |  | Hypothetical protein | *S. epidermidis* | AE015929 |
| 7 | 3668-3772 |  |  | 105 | 100 % | SE0049 |  | Hypothetical protein | *S. epidermidis* | AE015929 |
| 8 | 3760-3960 |  |  | 201 | 100 % | SE0050 |  | Hypothetical protein | *S. epidermidis* | AE015929 |
| 9 | 3760-4260 |  |  | 501 | 94 % | CA026 |  | Hypothetical protein | *MRSA* | FJ390057 |
| 10 | 4279-4587 |  |  | 309 | 100 % | CA027 |  | Hypothetical protein | *MRSA* | FJ390057 |
| 11 | 4592-4681 |  |  | 90 | 100 % | CA028 |  | Hypothetical protein | *MRSA* | FJ390057 |
| 12 | 4677-5024 |  |  | 348 | 100 % | CA029 |  | Hypothetical protein | *MRSA* | FJ390057 |
| 13 | 5528-7153 |  |  | 1626 | 100 % | *ccr*B4 |  | *ccr*B4 | *MRSA* | FJ390057 |
| 14 | 7153-8511 |  |  | 1365 | 100 % | *ccr*A4 |  | *ccr*A4 | *MRSA and*  *S. epidermidis* | FJ390057  AE015929 |
| 15 | 8701-9279 |  |  | 579 | 100 % | CA032/  SE0058 |  | Putative membrane protein/hypothetical protein | *MRSA and*  *S. epidermidis* | FJ390057  AE015929 |
| 16 | 9555-9671 |  |  | 117 | 100 % | ccrA/  SE0059 |  | Truncated *ccr*A | *MRSA and*  *S. epidermidis* | FJ390057  AM990992  AE015929 |
| 17 | 9789-10145 |  |  | 357 | 100 % | CA034/  SE0060 |  | Hypothetical protein | *MRSA and*  *S. epidermidis* | FJ390057  AE015929 |
| 18 | 10781-10876 |  |  | 96 | 100 % | CA035/  SE0061 |  | Hypothetical protein | *MRSA and*  *S. epidermidis* | FJ390057  AE015929 |
| 19 | 10999-12930 |  |  | 1932 | 100 % | copA |  | Copper-transporting ATPase copA | *MRSA and*  *S. epidermidis* | FJ390057  AE015929 |
| DR2^§^ | 13921-13939 |  |  | 19 |  |  |  |  |  |  |
| 20 | 14028-14654 |  |  | 627 | 99 % | SE0098 |  | Hypothetical protein | *S. epidermidis* | AE015929 |
| 21 | 14672-14911 |  |  | 240 | 100 % | SE0099 |  | Hypothetical protein | *S. epidermidis* | AE015929 |
| 22 | 15313-15441 |  |  | 129 | 96-99 % | Different MRSA and SE100 |  | Transposase | *MRSA and*  *S. epidermidis* |  |
| 23 | 15580-16161 |  |  | 582 | 99 % | SE101 |  | Transposase | *S. epidermidis* | AE015929 |
| 24 | 16267-17193 |  |  | 927 | 99 % | *arc*C |  | Carbamate kinase | MRSA USA300 and  *S. epidermidis* | CP000255  AE015929 |
| 25 | 17216-18211 |  |  | 996 | 99 % | *arc*B |  | Ornithine carbamoyl-transferase | MRSA USA300 and  *S. epidermidis* | CP000255  AE015929 |
| 26 | 18252-18938 |  |  | 693 | 99–100 % | SAUSA300  _0063/  SE0104 |  | cyclic nucleotide-binding domain protein | MRSA USA300 and *S. epidermidis* | CP000255  AE015929 |
| 27 | 18983-20401 |  |  | 1419 | 99 % | *arc*D |  | Arginine/ornithine  antiporter | MRSA USA300 and  *S. epidermidis* | CP000255, AE015929,  AF269944, AF269586 |
| 28 | 20490-21722 |  |  | 1239 | 99 % | *arc*A |  | Arginine deiminase | MRSA USA300 and  *S. epidermidis* | CP000255, AE015929,  AF269944,  AF269947,  AF269586 |
| 29 | 21994-22437 |  |  | 450 | 99-100 % | *arc*R |  | Arginine repressor | MRSA USA300 and  *S. epidermidis* | CP000255, AE015929,  AF269317,  AF269947 |
| 30 | 23525-23689 |  |  | 165 | 100 % | SE0108 |  | Type I restriction-modification system endonuclease | *S. epidermidis* | AE015929 |
| 31 | 23724-23816 |  |  | 93 | 100 % | SE0109 |  | Hypothetical protein | *S. epidermidis* | AE015929 |
| 32 | 23966-24394 |  |  | 429 | 99 % | SAUSA300_0067/SE0110 |  | Universal stress protein family | MRSA USA300 and  *S. epidermidis* | CP000255, AE015929,  AF269317,  AF269947, AF269442, CP000029 |
| 33 | 25279-25534 |  |  | 256 | 99 % | SE_0111* |  | Putative replication initiation protein Rep | *S. epidermidis* | AE015929 |
| 34 | 25618-26289 |  |  | 672 | 98 % | Several staphylo  cocci |  | IS431 transposase | Several staphylococci |  |
| DR3^§^ | 26484-26502 | DR3^§^ | 1-19 | 19 |  |  |  |  |  |  |
| 35# | 26726-27052 | 1# | 242-565 | 324 | No significant similarity |  | 100 % | transcriptional regulator | *S.epidermidis* | ZP04825861 |
| 36# | 27050-27192 | 2# | 563-708 | 140 | No significant similarity |  | No signifi  cant similarity |  |  |  |
| 37# | 27089-27922 | 3# | 605-1435 | 831 | No significant similarity |  | 100 % | Hypothetical  protein | *S.epidermidis* | ZP04825862 |
| 38# | 28127-28291 | 4# | 1643-1804 | 162 | No significant similarity |  | 100 % | Hypothetical  protein | *S.epidermidis* | ZP04825864 |
| 39# | 28597-28701 | 5# | 2110-2217 | 102 | No significant similarity |  | No signifi  cant similarity |  |  |  |
| 40# | 28608-29381 | 6# | 2124-2894 | 771 | No significant similarity |  | 100 % | Hypothetical  protein | *S.epidermidis* | ZP04825864 |
| 41 | 29426-29543 | 7 | 2942-3059 | 118 | 86 % | Truncated SAUSA300_0049^1^ |  | Hypothetical  protein | MRSA USA300 | CP000255 |
| 42 | 29622-29957 | 8 | 3138-3473 | 336 | 89 % | SAUSA  300_0050 |  | Hypothetical protein | MRSA USA300 | CP000255 |
| 43 | 30146-31090 | 9 | 3662-4606 | 945 | 99 % | SH0043 |  | Hypothetical protein, similar to 2-nitropropane dioxygenase | *S.haemolyticus* | AP006716 |
| 44 | 31229-31837 | 10 | 4745-5353 | 609 | 99 % | Truncated SH0044^2^ |  | Hypothetical protein, similar to LysR-gltR family transcription regulator | *S.haemolyticus* | AP006716 |

# Possible ORFs using ORF finder, Genbank. ^ The gene with the highest identity is listed. Only in cases where genes from several isolates have the same

identity, all are listed. *The last 5 bps of the gene are missing in the M1 sequence. ^1^Only the last 118 of 330 bps.  ^2^Only the last 612 of 867 bps.

^§^Possible direct repeats.
